# Survivin Inhibition by Piperine Sensitizes Glioblastoma Cancer Stem Cells and Leads to Better Drug Response

**DOI:** 10.3390/ijms23147604

**Published:** 2022-07-09

**Authors:** Neerada Meenakshi Warrier, Ramesh Kumar Krishnan, Vijendra Prabhu, Raghu Chandrashekhar Hariharapura, Prasoon Agarwal, Praveen Kumar

**Affiliations:** 1 Department of Biotechnology, Manipal Institute of Technology, Manipal Academy of Higher Education, Manipal 576104, Karnataka, India; neerada.m.w@gmail.com (N.M.W.); vijendra.prabhu@manipal.edu (V.P.); 2 Department of Medicine II, Hematology and Oncology, University Hospital Frankfurt, Goethe University, 60590 Frankfurt, Germany; rameshkumar.krishnan@dkfz-heidelberg.de; 3 German Cancer Consortium (DKTK) and German Cancer Research Center (DKFZ), 69120 Heidelberg, Germany; 4 Department of Pharmaceutical Biotechnology, Manipal College of Pharmaceutical Sciences, Manipal Academy of Higher Education, Manipal 576104, Karnataka, India; raghu.c@manipal.edu; 5 National Bioinformatics Infrastructure Sweden (NBIS), SciLifeLab, Division of Occupational and Environmental Medicine, Department of Laboratory Medicine, Lund University, 22362 Lund, Sweden

**Keywords:** glioblastoma cancer stem cells, therapeutic targeting, survivin, piperine, temozolomide, integrative analysis

## Abstract

Glioblastoma multiforme (GBM) cancer stem cells (GSCs) are one of the strongest contributing factors to treatment resistance in GBM. Identification of biomarkers capable of directly affecting these cells within the bulk tumor is a major challenge associated with the development of new targeting strategies. In this study, we focus on understanding the potential of the multifunctional extraordinaire survivin as a biomarker for GSCs. We analyzed the expression profiles of this gene using various publicly available datasets to understand its importance in stemness and other cancer processes. The findings from these studies were further validated using human GSCs isolated from a GBM cell line. In these GSCs, survivin was inhibited using the dietary phytochemical piperine (PIP) and the subsequent effects on stemness, cancer processes and Temozolomide were investigated. In silico analysis identified survivin to be one of the most significant differentially regulated gene in GSCs, in comparison to common stemness markers. Further validation studies on the isolated GSCs showed the importance of survivin in stemness, cancer progression and therapy resistance. Taken together, our study identifies survivin as a more consistent GSC marker and also suggests the possibility of using survivin inhibitors along with standard of care drugs for better therapeutic outcomes.

## 1. Introduction

Glioblastoma multiforme (GBM) is an aggressive grade IV astrocytoma. This is the most common, malignant and lethal brain tumor in adults and arises from multiple cell types including glial cells and various other neural stem-like cells [1]. The cells in the tumor are under different stages of differentiation from stem cells to neurons to glial cells [2]. They are usually highly potent primary tumors without a certainly known origin and are rarely less lethal when secondary, wherein low grade gliomas get converted following genetic manipulations [1,3]. It often remodels the tumor microenvironment with the help of the immune system, stroma and vasculature to survive [4]. These tumors display high inter and intratumor heterogeneity and mutations in various genes and signaling pathways [5]. Even though there are distinct subclassifications of GBM, intended to guide through diagnostic, prognostic and therapeutic recommendations, the majority of GBM patients receive identical treatments [6].

The current standard therapy for GBM includes maximal surgical resection, followed by concurrent radiotherapy, oral chemotherapy with temozolomide (TMZ) and adjuvant chemotherapy by TMZ [7]. Yet, the median post-diagnosis survival period is around 15–18 months, and the tumors tend to recur post treatment, developing into more aggressive ones, for which a standard of care therapeutic regimen is still not devised [8]. Resistance to therapy and dismal prognosis are attributed to the extensive invasiveness, the evasion of cell death, the protection of tumors by the blood–brain barrier (BBB), the intertumoral and intratumoral heterogeneity in GBM, the lack of dependable targets, its immunosuppressive nature, defective metabolism and the presence of GBM cancer stem cells (GSCs) [9,10,11]. As with any other tumor, the difficulty in identifying and targeting self-renewing GSCs present in the tumor is a contributing factor to treatment resistance.

A range of surface or internal markers such as cluster of differentiation (CD)133, CD15, SRY (sex determining region y)-Box 2 (SOX2), Nestin, oligodendrocyte transcription factor 2 (OLIG2), Nanog, octamer-binding transcription factor 4 (OCT4), Sal-like protein 4 (SALL4), L1 cell adhesion molecule (L1CAM), ATP binding cassette transporter G2 (ABCG2), etc., and signaling pathways have been used in targeting GSCs in bulk tumors. However, these are not unique for GSCs. Hence, the best option would be to target two or more co-expressed markers that have the potential to target multiple cancer stem cell (CSC) hallmarks [12,13,14].

The multifaceted extraordinaire survivin is a differentially expressed, oncofetal inhibitor of apoptosis protein that plays important roles in the regulation of cell division, cell death, tumor growth and maintenance, and CSC survival. The survivin gene Baculoviral IAP Repeat (BIR) Containing 5 (BIRC5) shows very little expression in normal adult differentiated tissues. Most cancers that show an increased expression of survivin are often associated with poor prognosis [15,16,17]. It is involved in signaling pathways, including but not limited to PI3K/AKT, JAK/STAT, WNT/β-Catenin, TGF-β, SMAD, NF-κB and NOTCH, in a variety of cancers [18]. Studies have reported the overexpression of this gene in GBM, where it mediates cell growth and maintenance, apoptosis, invasiveness, vascularization and therapy resistance and hence indicators of poor prognosis [16,19,20,21].

Small molecule inhibitors are the most well developed targeting molecules capable of directly or indirectly targeting survivin in cancers have already been suggested. They target survivin transcription, inhibit mitosis, protein−protein interaction, various upstream signaling pathways, etc. [15,22,23,24]. We looked into a range of phytochemicals with anticancer properties that are suggested in the literature.

Piperine (PIP) is an alkaloid from the dietary phytochemical Piper nigrum, more commonly known as black pepper. The anticancer mechanisms displayed by PIP include inhibition of apoptosis via caspase activation, cell cycle arrest at the G2/M phase and inhibition of signaling cascades including NF-κB, PI3K/AKT/GSK-3β, JAK/STAT3, WNT/β-Catenin, inhibition of angiogenesis, etc. [25]. There are studies suggesting its effect on CSC self-renewal, where it inhibited mammosphere formation by acting on NOTCH and WNT signaling pathways [26]. This drug was also reported to increase the bioavailability of standard of care drugs; in fact, it is the world’s first scientifically validated bioavailability enhancer. This dietary compound is capable of inhibiting drug-metabolizing enzymes P-gp and cytochrome P450 3A, responsible for first-pass elimination of many standard of care drugs [25,27]. Thus, PIP is capable of inducing cell death and increasing chemo and radio-sensitization in many cancers [28]. Molecular docking and in vitro studies together have suggested the potential of PIP as a direct survivin inhibitor. It is capable of triggering apoptosis by blocking PPI interaction of survivin with SMAC. It competes with SMAC and binds to the SMAC binding region in survivin, thereby making SMAC available to apoptosis. Studies have shown its effect on breast and colon cancer [29,30]. Molecular docking studies have showed it to bind to the protein with a binding energy −5.76 kcal/mol at similar active sites to which SMAC binds with a binding energy of −5.03 kcal/mol, thus unblocking apoptosis [31].

Though few, there have been reports suggesting the possibility of utilizing survivin as a potential therapeutic target in GBM. However, its importance in the GSC compartment is not well explored. Moreover, identification of strategies capable of directly targeting the markers is still an unresolved challenge. In this study, we compared the expression patterns of survivin with various other literature-curated stemness markers in the GSC compartment using various publicly available datasets. Then, we validated the findings on GSCs isolated from glioblastoma cell lines by using PIP to inhibit survivin in these cells and observed the effect on various cancer processes including self-renewal, proliferation, invasion, apoptosis and resistance to standard of care drug TMZ. Our study is aimed at identifying the potential of survivin as a therapeutic target in the GSC compartment and suggesting a novel therapeutic strategy for directly targeting this multifunctional protein, thereby improving the overall drug response in GBM.

## 2. Results

### 2.1. BIRC5 Is a Differentially Expressed Gene

To understand the expression pattern of BIRC5 in cancers, we obtained the expression profile of BIRC5 in 33 different tumors provided in the cancer genome atlas (TCGA) database and matching normal sample types from GETx database using GEPIA (Figure S1a). We found that this gene was almost always significantly differentially expressed between tumor and normal samples with higher expression in tumors, with acute myeloid leukemia being the only exception.

We looked specifically for the cell types with increased expression of BIRC5 using the webtool Stemformatics. Even though this was initially established just for stem cell types, currently it contains over 3000 cell types. We found that BIRC5 was expressed most predominantly by ESCs, iPSCs, endoderm cells, hematopoietic precursor cells, common myeloid progenitors and NPCs. We looked into multiple datasets in each cell type and have given a few reference plots in Figure S1b.

Next, we wanted to see if there is a change in its transcriptional levels in the normal brain, GBM and LGG specifically. For this, we studied the microarray (GSE4271 and Rembrandt) and RNA-seq (GSE48865, CGGA and TCGA-GBM-LGG) expression profiles of BIRC5 obtained from the GlioVis [32] portal for data visualization and analysis (Figure S1c). The normal brain showed negligible expression of the gene, whereas in LGGs the expression showed upregulation relative to the grade of the tumor (oligodendroglioma, oligoastrocytoma, astrocytoma, anaplastic oligodendroglioma, anaplastic oligoastrocytoma, anaplastic astrocytoma, glioblastoma and mixed glioma). This further confirmed the difference in transcriptional levels of BIRC5 within different grades of gliomas, with an upregulation in the grade IV gliomas or GBM.

### 2.2. BIRC5 Is Significantly Involved in Stemness in Cancer Cells

**Figure 1:**
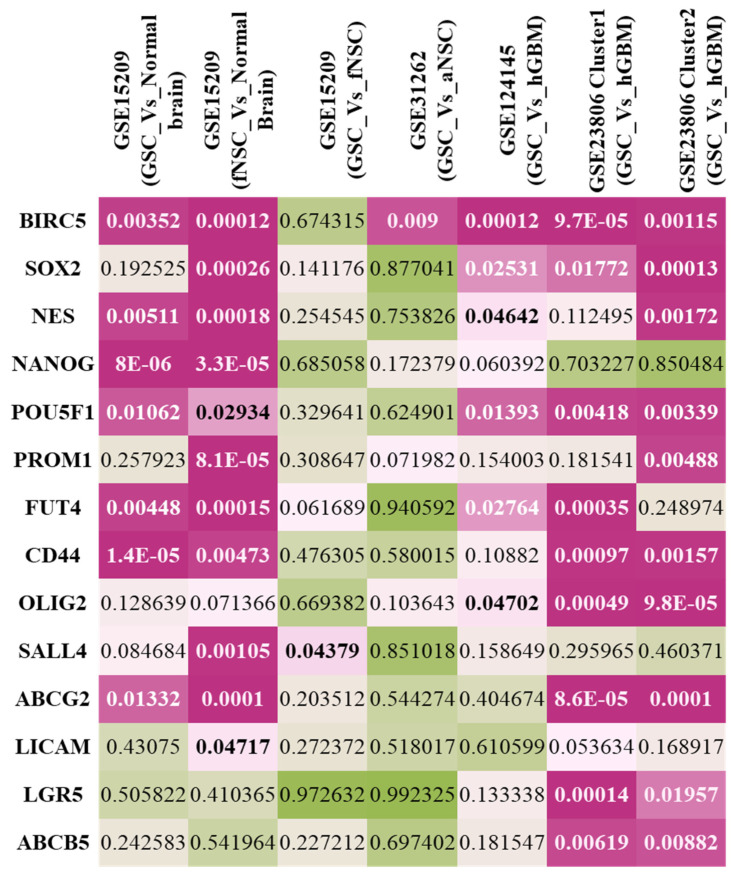
Heatmap showing the false discovery rate (FDR) values of differential expression of BIRC5 and the literature-curated stemness genes between samples of GSCs, hfNSCs, aNSCs, hGBM and normal brain. The adjusted *p*-values are indicated, and the significant ones (*p*-value < 0.05) are marked in bold.

**Figure 2:**
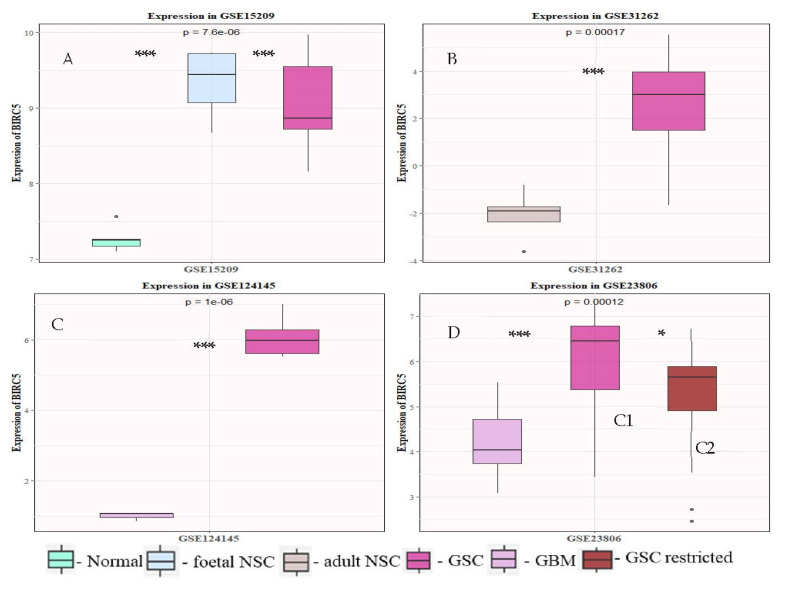
Differential expression of BIRC5 in GSC samples obtained from 4 different datasets: (**A**) GSE15209 showing expression in normal brain, GSCs and fNSCs; (**B**) GSE31262 for aNSC and GSC; (**C**) GSE214145 hGBM and GSC; and (**D**) GSE23806 primary GBM and GSC lines isolated from them. The graphical data are presented as mean ± SD, and the statistical analysis was performed using one-way ANOVA (*: *p*-value < 0.05; ***: *p*-value < 0.001).

Next, we wanted to see if BIRC5 was differentially regulated in GSC samples in comparison to bulk primary tumors, neural stem cells (NSCs; both adult and fetal) and normal brain tissues. For this, we collected the pre-processed microarray data of four different studies (GSE15209, GSE31262, GSE23806 and GSE124145) from the GEO database and compared the adjusted *p* values of BIRC5 with 13 literature-curated characteristic stemness genes commonly used for GSC identification and isolation (Figure 1). These included SOX2, Nestin, NANOG, POU5F1, PROM1 (CD133), FUT4 (CD15/SSEA), CD44, ABCG2, ABCB5, LGR5, L1CAM, SALL2 and OLIG2. We observed that BIRC5 was one of the most significant differentially regulated gene in GSCs in comparison to the other genes. Overall, we observed a significant change in the expression pattern of BIRC5 between the normal brain, human fetal NSCs (hfNSCs), adult NSCs (aNSCs), human GBM (hGBM) and GSCs. We also studied the level of expression of these genes in each of these samples and plotted them to get a better image of the patterns displayed (Figure 2 and Figure S2). We found that GSCs always showed statistically significant higher expression of BIRC5 unlike the normal brain, adult NSCs and hGBM samples. The only exception was fNSCs, which showed higher expression than GSCs. A similar pattern of expression was also displayed by SOX2 and OLIG2 in these studies, but both these genes were expressed highly in aNSCs. Further, NES and PROM1 were differentially expressed in GSCs when compared to the normal brain, but they displayed high expression in GBM samples. The other genes showed variations in expression patterns among different studies.

**Figure 3:**
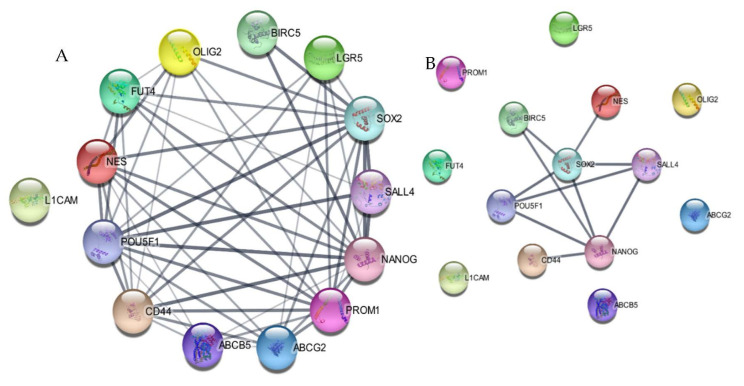
String analysis of the stemness genes using Cytoscape. (**A**) The protein−protein interaction (PPI) network of the genes with a confidence level of 0.4 with zero identifiers. (**B**) PPI network of the genes with a confidence level of 0.9 with zero identifiers, suggesting these genes to show significant interaction.

We were interested in finding out if BIRC5 interacted with any of these literature-curated stemness genes, and for this we performed a string analysis using Cytoscape. The analysis showed that all these genes, except L1CAM, interacted with each other without any identifiers. We found that BIRC5 interacted with SOX2 and NANOG, which were two of the genes that interacted with almost all other literature-curated genes at a confidence score of 0.04 (Figure 3A). To check for possible interaction with a more stringent cut-off, we analyzed the interaction at a confidence score of 0.90 and found that SOX2, Nestin, NANOG, BIRC5, POU5F1, CD44 and SALL4 formed a network (Figure 3B).

### 2.3. BIRC5 Was Correlated to Stemness and Proliferation Genes

**Figure 4:**
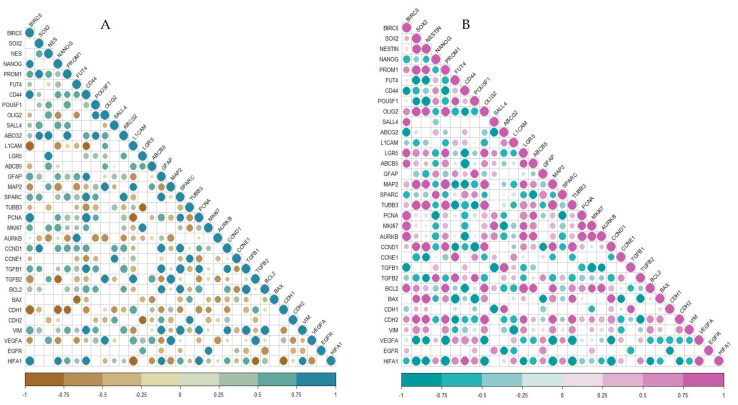
Correlogram of genes from GSE15209. (**A**) Normal; (**B**) GSC. The size of the circle indicates the significance of the *p*-values; non-significant ones are blank with no circles.

**Figure 5:**
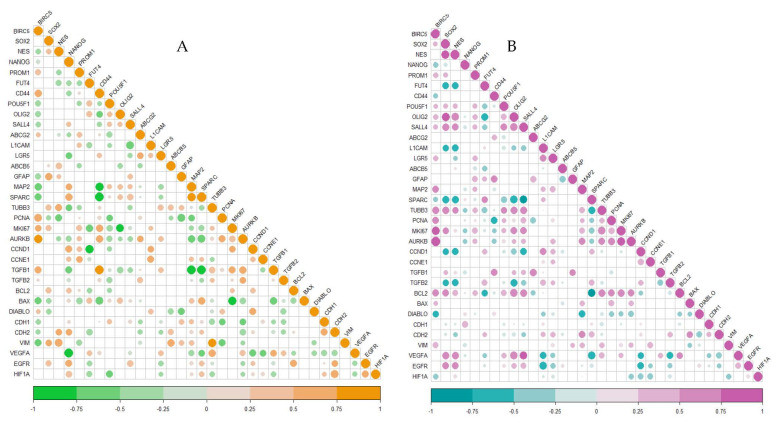
GSE23806. (**A**) GBM; (**B**) GSC. The size of the circle indicates the significance of *p* values; non-significant ones are blank with no circles.

To understand the correlation of BIRC5 with the stemness genes and literature-curated genes involved in the cancer initiation, progression and maintenance, we studied the correlation patterns of these genes from all the previously mentioned datasets (Figure 4 and Figure 5). Other than the stemness genes mentioned before, we looked into differentiation markers (GFAP, MAP2, SPARC and TUBB3), proliferation markers (PCNA, MKI67, AURKB, CCND1 and CCNE1), genes active in apoptosis (DIABLO, BCL2, BAX, TGFβ1 and TGFβ2), migration markers (CDH1, CDH2 and VIM) and angiogenesis markers (VEGFA, EGFR and HIF1A). Our analysis showed that BIRC5 was significantly positively correlated to various stemness genes including SOX2, Nestin, PROM1, POU5F1, OLIG2, SALL4, etc., and proliferation markers PCNA, MKI67 and AURKB in GSC samples in almost all datasets. Interestingly, its correlation to some stemness markers—mainly Nestin, POU5F1, OLIG2 and SALL4—showed a negative correlation to BIRC5 in both normal brain samples and GBM, whereas in GSC they displayed a positive correlation. We also observed variation in its correlation with differentiation markers GFAP, SPARC and MAP2 among normal brain and GSC and also GBM and GSC. However, BIRC5 always showed a positive correlation with proliferation markers, ascertaining its role in the process.

### 2.4. Differential Trypsinisation of U87 Cells Generated Three Pools of Cells with Varying Sensitivity to Trypsin

Subjecting 60–70% confluent cells to varying concentrations of trypsin, two different subpopulations of cells—S (which was sensitive to trypsin) and R (which was resistant)—were isolated. To obtain a better separation between the more adherent and less adherent cells, we followed the second of the two protocols suggested by Morata-Tarifa et al. [33], where a population of cells with adhesion greater than S but less than R was removed. In order to understand the functional characteristics of the isolated subpopulations and their stem cell properties, we performed a variety of stem cell assays on these cells, keeping the entire population of cells, P, as control. All experiments were performed in triplicate and were repeated for at least two biological replicates.

### 2.5. The Trypsin-Sensitive Cells Displayed Higher Self-Renewal Potential

**Figure 6:**
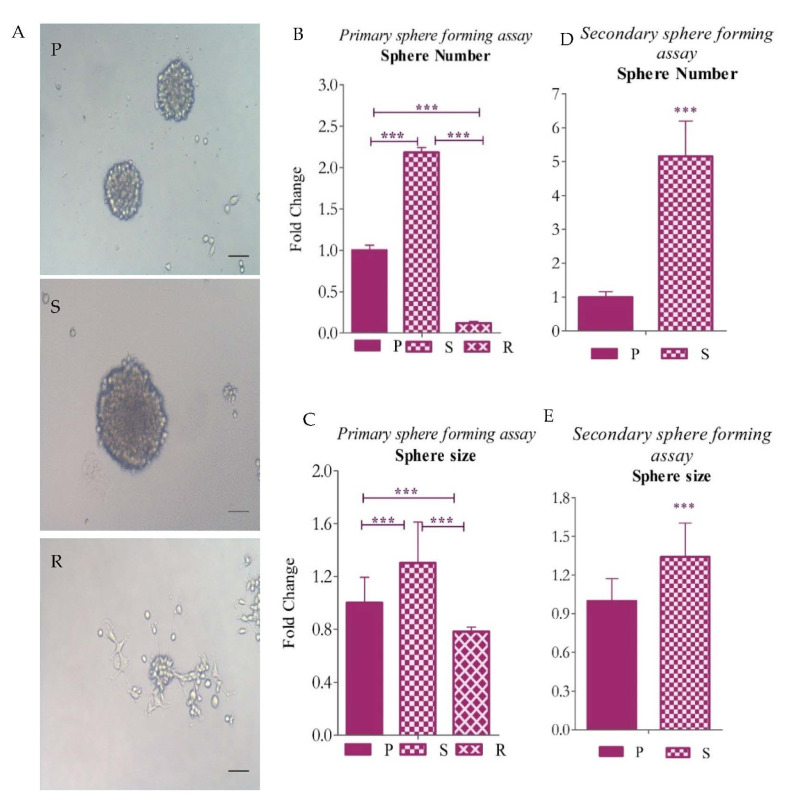
Morphology and sphere number and size of isolated and enriched CSCs in U87 cells. (**A**) Morphology of spheres of P, S and R, respectively, after 6 days of incubation in non-adherent culture conditions; (**B**,**C**) sphere number and size of spheres in primary sphere-forming assay; and (**D**,**E**) sphere number and size of spheres in secondary sphere-forming assay. The graphical data are presented as mean ± SD, and the statistical analysis was performed using one-way ANOVA (***: *p*-value < 0.001). The scale bar indicates 20 µm.

The isolated subpopulations grown in non-adherent tissue culture plates, supplemented with SFM and containing growth factors, were observed after 6 days of incubation (Figure 6). We counted the spheres > 70 µm in size and measured the size of the spheres for a better understanding. We observed that the S population grew as spheres in anchorage-independent conditions. The number and the size of these spheres showed a significant increase in comparison to R and P cells (Figure 6B,C). R cells formed very few small spheres to no spheres in these conditions, and some were also found to display adherent growth patterns, whereas the P cell showed a mixed pattern of growth (Figure 6D,E). We also performed a secondary sphere-forming assay post 70–80% confluency, achieved in the primary spheres. The spheres were dissociated using trypsin and replated at a proportion of 500 cells per well. After 6 days of incubation, we observed that the S cells were still forming spheres that resembled the primary spheres. The R cells were not able to propagate as spheres further under the culture conditions, whereas P displayed a reduction in the number and size of spheres formed in comparison to the primary cultures (Figure 6).

We continuously cultured the S cells in SFM for about five passages to confirm their ability to continuously self-renew and proliferate. We observed that these cells were able to proliferate in this medium for the multiple number passages, further adding to the increased presence of stem cell phenotypes in them.

### 2.6. The Sensitive Population of Cells Displayed Clonogenic Potential

**Figure 7:**
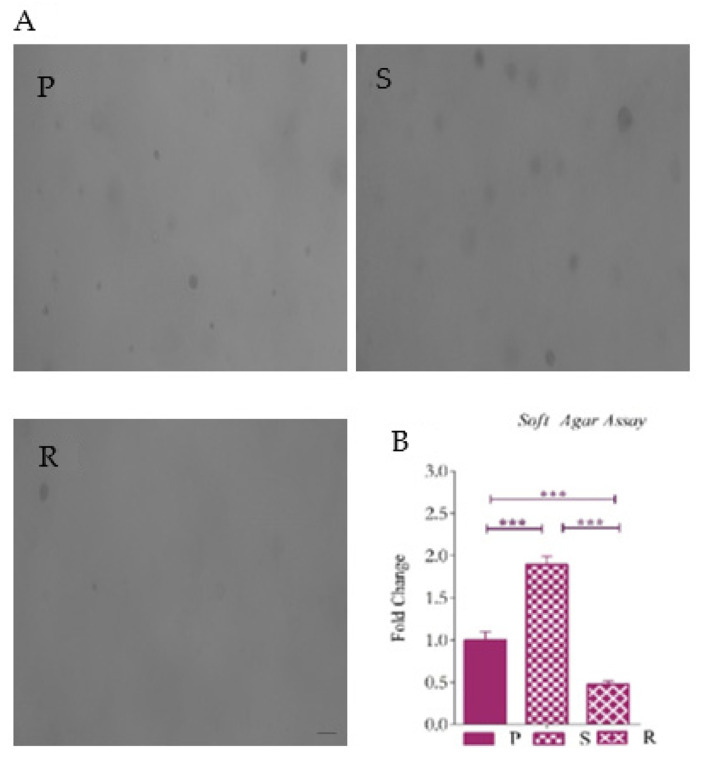
Soft agar colony-forming assay. Representative images of (**A**) P cells, S cells and R cells in soft agar. (**B**) Graph of the fold change of the number of colonies counted after 15 days of incubation. The graphical data are presented as mean ± SD, and the statistical analysis was performed using one-way ANOVA (***: *p*-value < 0.001). The scale bar indicates 20 µM.

To understand the colony-forming potential of the enriched cells in anchorage-independent conditions, we performed the gold standard soft agar assay. After incubating the same number of cells in soft agar for 25 days, we observed that the trypsin-sensitive pool of cells displayed better clonogenic potential in comparison to the other two. It was observed that the colonies formed by S cells were bigger in size and number. Single cells incapable of self-renewal were observed in the R pool. These cells showed very few colonies. However, most colonies were very small (Figure 7). The data were in concordance with the ability to form spheres in these cells and further confirmed the presence of CSCs in the S pool and their absence the R pool.

To further confirm the self-renewing potential of these cells, we looked for the ability of single cells of each type to form spheres in non-adherent growth conditions. The percentage efficiency of colony formation was found to be 28.57%, 66.67% and 3.09% in P, S and R, respectively. The significant increase in sphere size was observed in these single cell colonies from S population as well. The resistant pool of cells formed spheres, but their smaller size suggested the inability of these cells to multiply continuously.

### 2.7. The Trypsin-Sensitive Population of Cells Showed Better Proliferation and Migration Potential

**Figure 8:**
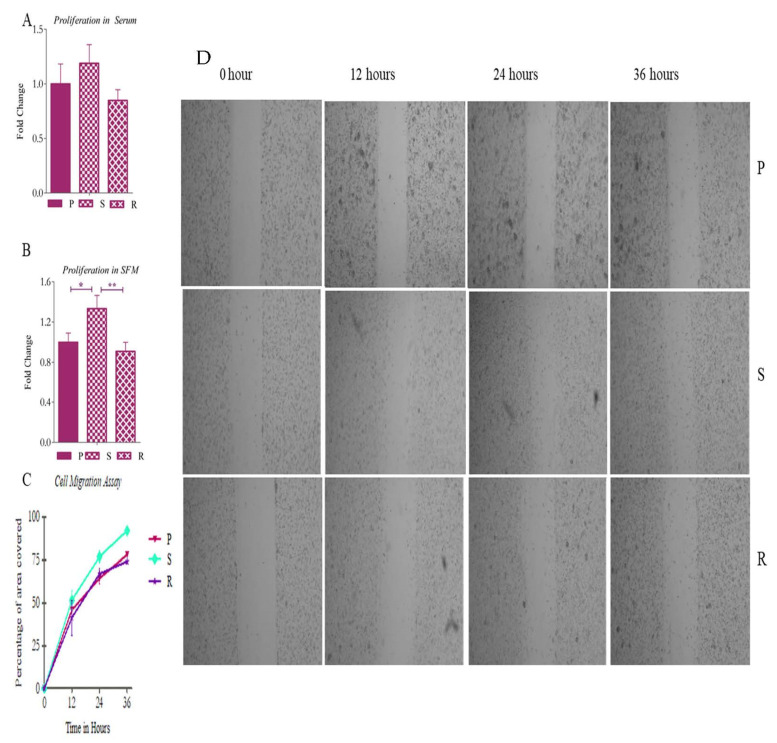
Proliferation and migration assay. (**A**) Proliferation of the isolated pools in serum-containing media and (**B**) serum-free media. (**C**) The percentage migration in P, S and R cells in the in vitro wound healing assay. (**D**) Representative images of P, S and R cells at 0 h, 12 h, 24 h and 36 h. The graphical data are presented as mean ± SD, and the statistical analysis was performed using one-way ANOVA (*: *p*-value < 0.05; **: *p*-value < 0.01).

Next, we studied the proliferation potential of these cell types in both serum-containing and serum-free media (Figure 8A,B). It was observed that the S cells showed no significant proliferation potential in adherent growth conditions in comparison to P and R cells. There was no significant difference in proliferation between the P and R populations. However, in SFM, these cells showed an increased rate of proliferation compared to the other pools. This added to the existing evidence supporting the presence of a more CSC-like phenotype in the S pool.

We performed in vitro wound healing assay [34] to understand migration in these cells. The cells grown in monolayer were scratched, and after 12, 24 and 36 h in serum starved conditions, the data were collected. We quantified the migration using the area method. The area of the gap that was covered at the time points was 46%, 64% and 78%, respectively, in P; 52%, 77% and 92%, respectively, in S; and 41%, 67% and 74%, respectively, in R cells (Figure 8C,D).

### 2.8. The Trypsin-Sensitive Pool Expressed Stemness Markers

**Figure 9:**
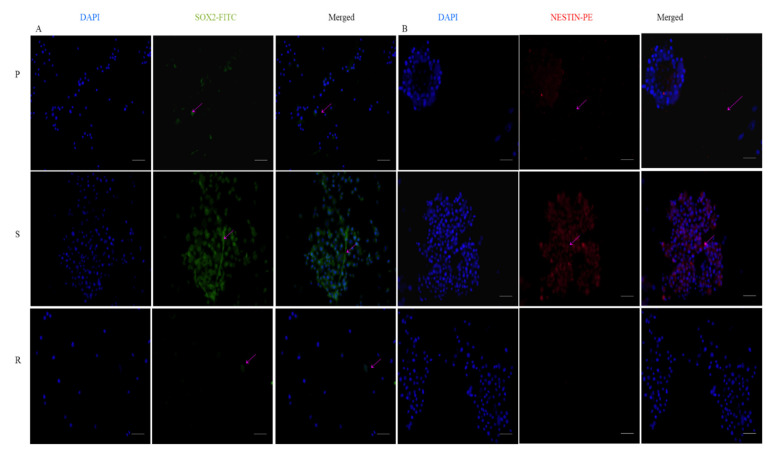
Expression of stemness markers: immunocytochemistry images of (**A**) Sox2 primary antibody stained with FITC and counterstained with DAPI; (**B**) Nestin-primary antibody stained with PE and counterstained with DAPI. The scale bar indicates 20 µm.

Immunostaining or immune cytochemistry was our next step in confirming the presence of CSC-like phenotype in the cell pools. We looked for the expression of two well-known stemness markers—SOX2 and Nestin—in P, S and R populations (Figure 9A,B). We were able to detect a significant expression of both of the markers in the sensitive pool, but they were negligible in the resistant ones. P expressed the antibodies of both markers, but less than S. The fluorescent intensities have been provided in Figure S3A.

**Figure 10:**
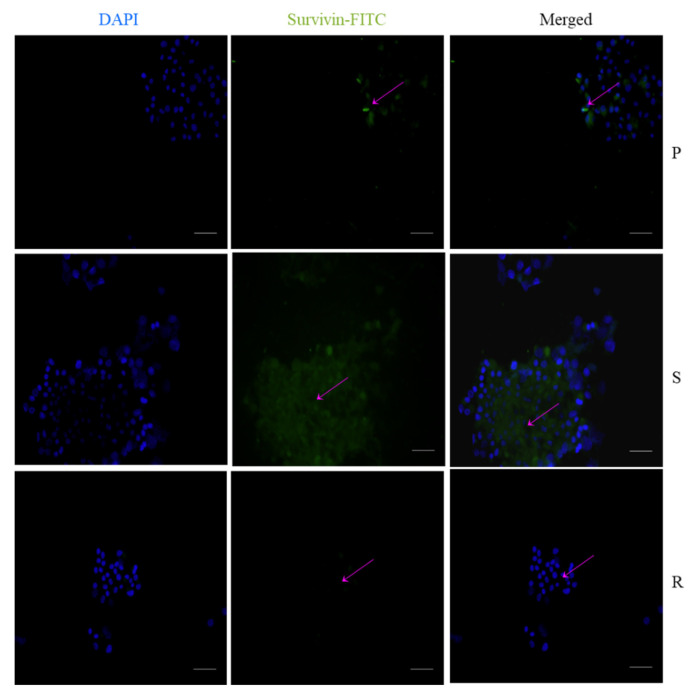
Expression of survivin by immunocytochemistry; survivin primary antibody stained with FITC and counterstained with DAPI. The scale bar indicates 20 µm.

We also looked into the expression of survivin in these cells, and our observation suggested that the S pools displayed higher expression of survivin (Figure 10). The fluorescent intensities have been provided in Figure S3B.

Overall, the S pool isolated by differential trypsinization displayed stem-like properties in comparison to the total population P and the trypsin-resistant R. The R pool did not display any stem cell properties; hence, we removed the R pool from the rest of the analysis.

### 2.9. Piperine at very Low Concentration Inhibits Survivin in Both P and S Cells

**Figure 11:**
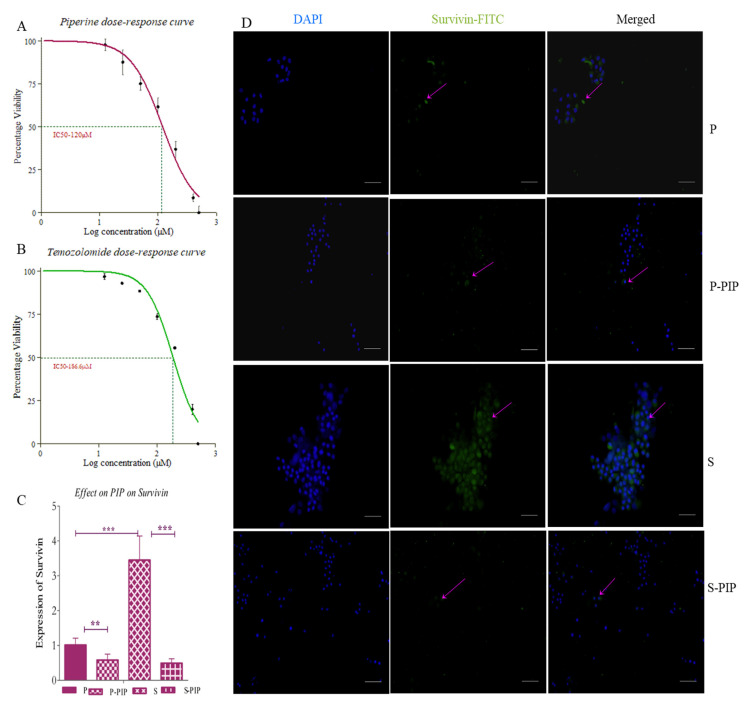
Inhibitory effect PIP on survivin: (**A**) dose−response curve of PIP and (**B**) TMZ on U87 cells. (**C**) The inhibition of survivin by PIP. Expression of survivin in control and treated cells studied using RT-PCR. (**D**) Immunostaining images of survivin on both P and S cells with and without PIP treatment, stained using FITC and counterstained with DAPI. The graphical data are presented as mean ± SD, and the statistical analysis was performed using one-way ANOVA (**: *p*-value < 0.01; ***: *p*-value < 0.001). The scale bar indicates 20 µm.

To identify the inhibitory effect of PIP in the cell line, we treated the cells with 25 µM (~ IC10) PIP for 48 h. The concentration was selected based on the IC50 value of PIP, which was 120 µM (Figure 11A). After this, both the treated and control cells were analyzed for the expression of survivin, using both IHC and RT-PCR. The gene expression analysis showed around 75% reduction in survivin expression in the S population and about 50% in the P cells (Figure 11C). The IHC results were also similar to this (Figure S4). Our results pointed towards the potential PIP as an inhibitor of survivin in both GBM and GSCs. It was observed that PIP affected survivin expression in S cells more than in that of P cells (Figure 11D).

### 2.10. Survivin Inhibition Increases the Efficacy of Standard of Care Drugs

**Figure 12:**
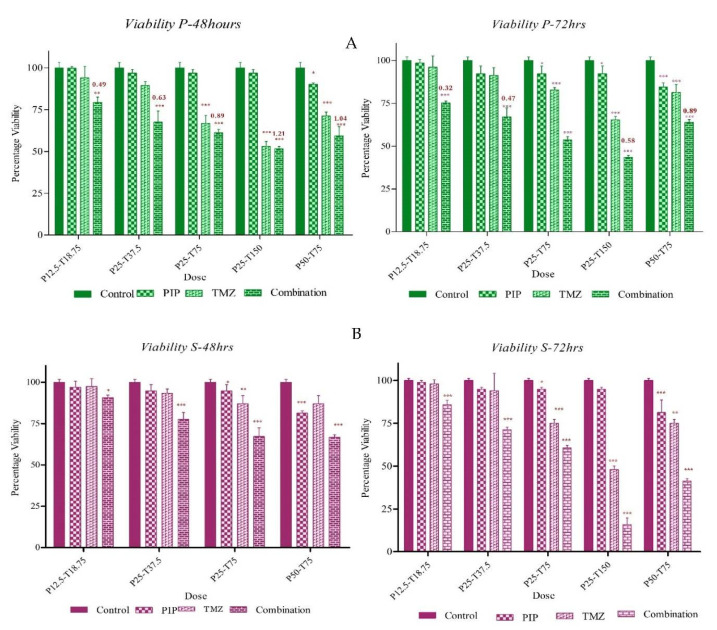
The effect of survivin inhibition on P and S cells. The effect on the efficacy of TMZ. (**A**) The percentage viability with single and combination drugs for 48 and 72 h for P and (**B**) for S cells. The CI values are indicated above the graph of P cells. The graphical data are presented as mean ± SD, and the statistical analysis was performed using one-way ANOVA (*: *p*-value < 0.05; **: *p*-value < 0.01; ***: *p*-value < 0.001).

To understand the effect of inhibiting survivin using PIP on the standard of care drug TMZ, we first studied the effect of different concentrations below the IC50 (120 µM for PIP and 186.6 µM for TMZ; Figure 11A,B) values of both the drugs as single agents, and then compared it with drug combinations and expressed it as combination index (CI). We performed the assay for 24 h, 48 h and 72 h. Although 24 h of treatment with the drug had no significant effect alone or in combination, 48 h and 72 h showed significant effects. The CI values indicated that the drug combination has a synergistic effect at lower concentration of both drugs. Hence, we can state that PIP inhibits survivin at a very low concentration and increases the efficacy of the standard of care drugs. We also found that the combination has a very significant effect on the viability of both P (Figure 12A) and S cell pools (Figure 12B) where the single drug has very little effect. The CI value of the combination is indicated on top of the graph of P cells. This indicates the involvement of survivin in resistance to first line therapy.

### 2.11. Effect of Survivin Inhibition on Stemness

**Figure 13:**
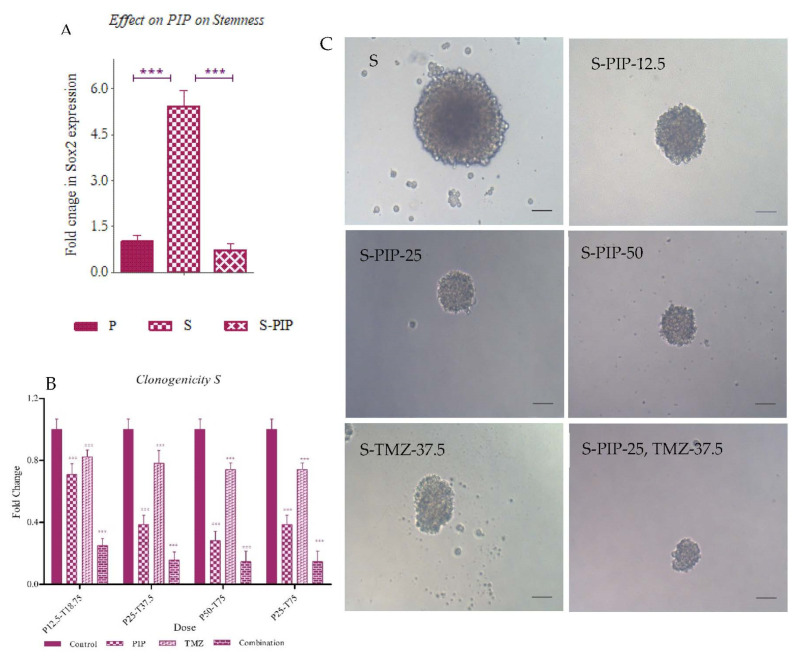
Effect of survivin inhibition on stemness. (**A**) The expression of stemness marker Sox2 in PIP treated cells using RT-PCR (**B**) Clonogenic potential in the isolated GSCs with single drug and combination treatments. (**C**) Representative images of colony formation in the S pool with and without treatment. The graphical data are presented as mean ± SD and the statistical analysis was performed using one-way ANOVA (***: *p*-value < 0.001). The scale bar indicates 20 µm.

To understand effect of the treatment on stemness, we studied the expression of stem cell marker Sox2 when treated with 25 µM PIP and also the clonogenic potential of the cells with both single and combination treatments. We found that the S cells showed a six-fold increase in the expression Sox2 in comparison to P cells, but upon PIP treatment, the level of expression was reduced by almost eight-fold (Figure 13A).

Clonogenic assay observations were taken after 12–14 days of incubation post-treatment with PIP and TMZ, and the combination suggested that PIP treatment had a very significant effect on reducing the number of colonies in S cells (Figure 13B,C). TMZ did not show much reduction in colony number whereas the combination did, which we can suggest could potentially be the effect of PIP. It was observed as well in both P and S pools. These findings supported the evidence of the role of survivin in stemness.

### 2.12. Effect of Survivin Inhibition on Invasion

**Figure 14:**
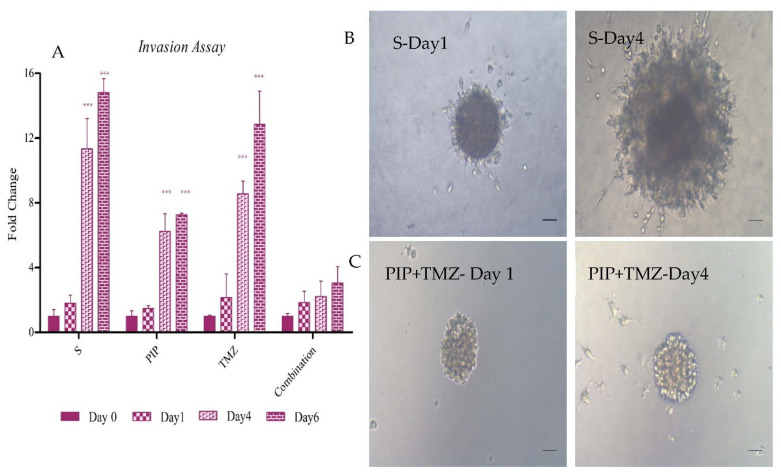
The effect of survivin inhibition on invasion. (**A**) The effect of PIP, TMZ and combination on invasion in the GSC population from day 0 to day 6. (**B**,**C**) Representative images of migrating spheres on Geltrex. (**B**) Images for control cells on Day 1 and Day 4, respectively. (**C**) The images of combination of 25 µM PIP and 37.5 µM TMZ on Day 1 and Day 4, respectively. The graphical data are presented as mean ± SD, and the statistical analysis was performed using one-way ANOVA (***: *p*-value < 0.001). The scale bar indicates 20 µm.

To study the effect of the treatments on invasion on GSCs, we used an invasion assay performed on extracellular matrix. We observed that, after 6 days of incubation, the control cells showed a 15-fold change in the size of the initial sphere plated. The PIP-treated cells showed a six-fold increase in the size, whereas TMZ alone showed invasive potential, which was almost similar to that of control cells. However, the interesting fact was that the combination treatment of the drugs did not show any significant migration on day 6 in comparison to day 1. The change in invasive potential on treatment with PIP alone and the ineffectiveness on treatment with TMZ further confirmed the role of PIP in reducing the migratory potential of cells (Figure 14).

### 2.13. Effect of PIP and TMZ on Apoptosis

**Figure 15:**
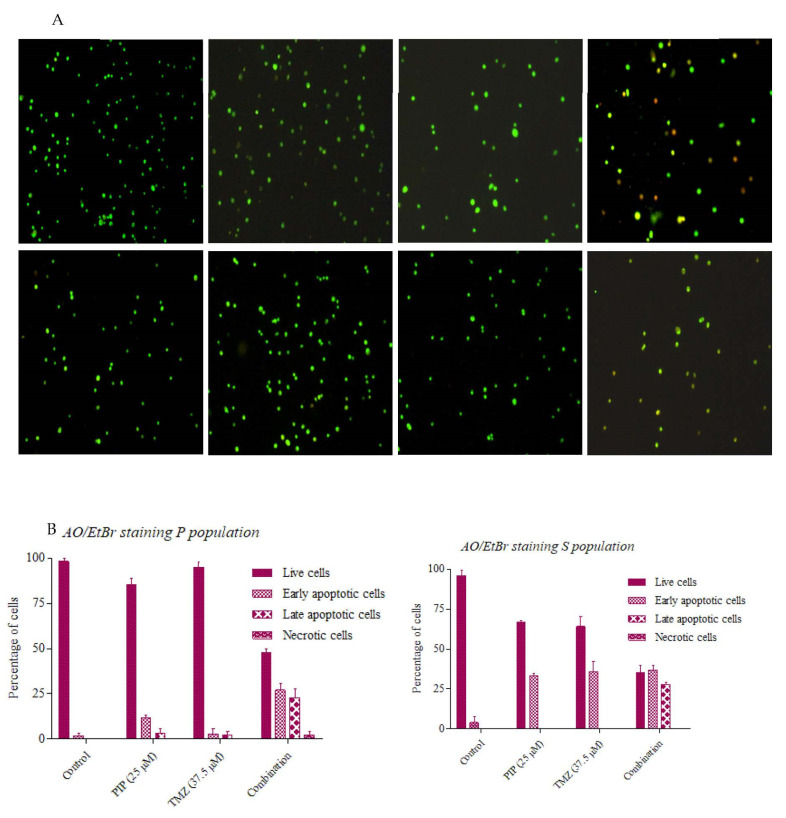
Effect of PIP and TMZ on apoptosis. (**A**) Fluorescent images of AO/EtBr staining of both P and S populations with and without treatment. (**B**) Quantitative analysis of the percentage of average number of live, early apoptotic, late apoptotic and necrotic cells in P and S populations with and without treatment.

Acridine orange (AO)/ethidium bromide (EtBr) is a simple fluorescent staining technique involving AO and EtBr to detect apoptosis in cells. Upon treatment, AO is taken up by both live and dead cells and emits green fluorescence upon binding to DNA and red on binding to single stranded RNA, and EtBr is taken up only by dead cells emitting red color. Morphologically, live cells and early apoptotic cells emit green fluorescence, but the viable ones display uniform bright green nuclei with an organized structure, whereas early apoptotic cells have bright green patches of perinuclear chromatin condensation along with the green nuclei. Late apoptotic and necrotic cells both emit red fluorescence, but the former display a nucleus with condensed or fragmented chromatin and the latter, one with an organized structure, resembling the viable cells (Figure 15).

Our results were relatively similar. The control cells P and the GSC population displayed a uniform nucleus displaying no significant apoptosis. The cells treated with PIP and TMZ alone few had early apoptotic cells, whereas the cells with combination treatment showed some late apoptotic cells, and we did not observe many necrotic cells within the suggested time (48 h) of treatment.

## 3. Discussion

GBM is the most aggressive form of brain tumors that tends to recur post treatment, developing into more aggressive ones [8]. The lack of specific markers with differential expression profiles in normal brain, GBM and GSCs is one of the major challenges encountered in developing potential therapeutic strategies capable of directly targeting GSCs in bulk tumors. In this study, we investigated the potential of survivin as a GSC marker and investigated the possibility of targeting this protein directly using dietary phytochemical PIP, with the aim of sensitizing the GSC population to the standard of care drug temozolomide.

We first conducted an extensive integrative analysis of publicly available microarray and RNA seq datasets of the normal brain, fNSCs, aNSCs, LGG, GBM and GSCs to understand the expression patterns of this gene. We found that BIRC5 was differentially expressed in normal and tumor samples in 32 out of the 33 cancer types provided in the TCGA database. We also found this gene to be highly expressed in various stem cell types as well. Previous studies on survivin have reported the expression of this protein to be high in tumors and stem cell types and also suggested its role in teratoma formation [35,36,37,38]. Our analysis of GSC datasets showed this gene to be one of the most differentially expressed genes in GSCs in comparison to 13 literature-curated stemness genes. GSE15209 contained expression data for human fNSCs and GSCs, propagated using growth factors EGF and FGF in adherent culture conditions resulting in homogeneous populations of stem cells and also from normal brain samples [39]. We observed a significant change in BIRC5 expression in GSC and fNSC in comparison to normal brain, but not in comparison to each other. Other stemness markers which showed similar patterns were Nestin, Nanog, Oct4, CD15, CD44 and ABCG2. We then wanted to see if there is variation in its expression in GSCs when compared to adult NSCs, and for this we used GSE31262 [40]. Interestingly, BIRC5 was the only gene among the set of genes that showed significant fluctuation in its expression. We next studied the change in expression profiles between GSCs and human primary GBM. For this, we first looked into GSE124145, where we compared the expression changes between patient-derived GBM samples and human GSC line X01 and X03 and found that BIRC5 was the most significant differentially expressed gene within our panel [41]. We further looked into GSE23806, a relatively large dataset with 12 primary GBM samples, the GSC lines at various passages established from them in serum-free conditions, and four monolayer cultures established from the same tumors as GS-lines and 32 conventional glioma cell lines [42,43]. We were interested in the 12 GSC lines established under serum-free conditions that were categorized in two clusters, Cluster 1 with stem cell phenotypes and proneural gene expression signature and Cluster 2 with restricted stem-like character and mesenchymal expression signature and the matched primary tumor samples. Here, once again, we found that BIRC5 was most significantly differentially regulated in the cluster with stem cell phenotypes and was also differentially expressed in the GSC population with restricted stemness in comparison. Analyzing the expression profiles of the literature-curated stemness genes showed that SOX2, Nestin and OLIG2 showed expression patterns similar to BIRC5 in these GSC datasets. [44,45,46]. The string analysis of these genes suggested that they form a closely linked PPI network, and BIRC5 interacts with SOX2 and Nanog. Our correlation analysis further supported this as we could see BIRC5 was positively correlated with SOX2, NES, PROM1, OLIG2 and SALL4 in GSCs and not in normal or GBM samples. With NANOG, it showed a negative correlation in GSCs and GBMs, whereas a positive correlation was seen in normal samples. BIRC5 showed a negative correlation to CD44 in GSC, whereas in normal and GBM it was positive. The correlations of these genes to BIRC5 in GSCs has not been explored thoroughly, though few studies have suggested possible interactions between some of them [47,48,49,50]. These results further confirmed the significance of BIRC5 as a GSC marker and its advantages with respect to other widely used markers. Interestingly, BIRC5 was found to be highly positively correlated to proliferation markers, more so in GSCs than GBM or normal, further confirming their role in the proliferation of the stem cell phenotypes in tumors.

Our next objective was to validate the findings from the integrative analysis in vitro. For this, we first isolated GSCs from the U87 cell line using differential trypsinization. The method is an extension of the established culturing method for epithelial cells and is closely associated with the ability of these cells to undergo a transition from epithelial to mesenchymal phenotype, a prominent feature of CSCs [51,52]. The protocol differentiates cells to more stem-like and less stem-like cells based on their adherence to the surface of the culture dish. The isolated S population consists of cells that detach quickly on treatment with very low trypsin concentration, although the R pool consists of cells that are significantly different from the S population in terms of anchorage. With differential trypsinization, we were able to enrich cells displaying stem cell phenotype in U87. These isolated and enriched cells could directly be used for downstream applications such as identification of biomarkers, screening for drugs, developing strategies targeting CSCs, etc.

We then characterized the isolated cell pools based on their ability to self-renew. The ability of a small number of cells or a single cell to form spheres under a serum-free condition is indicative of the self-renewal potential in these cells. Another standard method for understanding self-renewal and anchorage-independent growth potential is the soft agar assay to look for the ability to form holoclones [53,54,55]. Our observation pointed towards the higher self-renewal and improved anchorage-independent growth in the S subpopulation in comparison to P or R subpopulations, suggesting the presence of more CSC-like phenotypes in this population. The wound healing assay confirmed the migratory potential of the isolated GSCs. This could be attributed to the increased EMT in these cells. The expression of stemness markers is one of the most promising methods to authenticate the presence of stem cells population. We used some of the most promising stem cell markers for this purpose and found that the S cells expressed both Nestin and SOX2 with intensities higher than that of P, whereas the R pool showed minimal expression. We further wanted to look for the expression of survivin in these cells. We found survivin expression to be high in the cells with GSC phenotype, and the R pool showed lower expression. We further cultured the S pool in SFM and used these GSCs for understanding the role of survivin.

We used PIP, a known survivin inhibitor as per molecular docking reports, to inhibit survivin in these cells and study the effects. PIP is a common dietary phytochemical that has a binding affinity for the SMAC binding site of survivin [31], thus making SMAC available for apoptosis. We found that PIP has a dose-dependent effect on the U87 cell line, with an IC50 value of 120 µM. It was observed that the drug at a lower concentration had a negligible effect on cell viability. RT-PCR analysis of the treated and nontreated cells for the expression of survivin showed that the compound was capable of inhibiting survivin, even at lower concentrations. We confirmed this with immunocytochemistry as well and found similar results. This suggested the potential of PIP as a survivin inhibitor in GBM and GSCs.

Next, we wanted to see the effect of PIP on the standard of care drug for GBM, TMZ. TMZ also had a dose-dependent effect on GBM cells and U87 was not resistant to TMZ. The IC50 value of the drug was found to be 186.6 µM, but this drug as well did not show any effect on viability at a lower concentration. The combination of PIP and TMZ at lower concentrations was found to have a synergistic effect on the U87 cell line, suggesting that PIP enhances the efficiency of TMZ. This further adds to the already existing fact that PIP is a bioavailability enhancer of standard of care drugs [25].

Our, next aim was to understand the impact of inhibiting survivin in the stemness of the GSCs. PIP treated GSCs showed reduction in colony-forming potential. More interestingly, we observed that TMZ at low concentrations, as a single drug, failed to have a prominent impact, but when combined with PIP showed substantial reduction in the self-renewing potential of GSCs. We also studied the expression profile of stemness marker SOX2 in the treated cells and found a considerable reduction in the expression post treatment. This further ascertains the importance of survivin in stemness and also the inhibitory potential of PIP. We also observed that the PIP-treated GSCs displayed a significant reduction in the migratory potential of cells. The combination-treated cells showed very negligible movement after six days of incubation. Our results suggested PIP as a good inhibitor for direct targeting of GSCs via survivin.

Next, we wanted to see the effect of treatment on apoptosis induction. AO/EtBr staining results showed that the treatment at low concentrations of both PIP and TMZ did not induce cell death. However, we saw both early apoptotic and late apoptotic cells in combination treated the population. There were few necrotic cells in this population as well, suggesting that the treatment triggers apoptosis in both GBM and GSCs. This contributes towards the possibility of using the combination of PIP and TMZ as a new treatment strategy in GBM. Importantly, as evident from our integrative analysis, survivin is not GBM-specific; therefore, survivin inhibitors can be used in other tumor types along with their respective standard of care drugs.

## 4. Materials and Methods

### 4.1. Expression Profiles of BIRC5

#### 4.1.1. Differential Regulation across All Cancers

To understand the expression of BIRC5 and to deduce its importance in cancer versus normal samples, we compared the expression profile of the gene in 33 different TCGA tumor types to matched normal samples from the GTEx database using the interactive webtool GEPIA (http://gepia.cancer-pku.cn/ (5 July 2022)) [56]. Here, we used the Expression DIY module to obtain a dot plot of the expression of BIRC5 across 9736 tumors and 8587 normal samples. ANOVA was used as the differential method, the q-value cut off was set at 0.01 and log scale was chosen for the plot.

#### 4.1.2. Expression in Stem Cell Types

Studying the expression of the BIRC5 in stem cell types was the next step to deciphering the gene. For this, we used the web tool Stemformatics (https://www.stemformatics.org/ (5 July 2022)), which encompasses more than 300 different microarray, RNA-seq and scRNA-seq datasets with information on more than 3000 cell types [57,58]. We used the ‘gene to sample’ tool in the Gene module with tool as ‘cell types’ and looked for the expression of BIRC5 in various cell types across many datasets. The highly expressed cell types of BIRC5 were identified, and the log2 expression plots were downloaded from the site.

#### 4.1.3. Expression in GBM

We also looked for the differential expression patterns of BIRC5 in different types of GBMs. We used GSE4271 (24 LGG and 76 GBM samples) and Rembrandt (28 normal, 225 LGG and 219 GBM samples) for microarray data and TCGA RNA Seq data (10 normal, 515 LGG and 156 tumor samples), CGGA (625 LGG and 388 GBM samples) and GSE48865 (174 LGG and 100 GBM samples) for RNA-seq data obtained from Gliovis for expression analysis [32].

#### 4.1.4. Expression in GSCs

Next, we specifically wanted to see if BIRC5 was differentially regulated in GSCs. Here we explored four publicly available microarray datasets—GSE15209, GSE31262, GSE124145 and GSE23806—from the GEO database. GSE15209 contains the expression profiles of human fetal NSCs (fNSCs), GSCs and normal brain samples from adult human cortex. Data pre-processed using the vsnrma method from the Bioconductor package VSN were downloaded and annotated using GPL570_[HG-U133_Plus_2]_Affymetrix Human Genome U133 Plus 2.0 Array [39]. GSE31262 reported the gene expression profiles of 5 individual samples of human adult NSCs (aNSCs) and 9 individual samples of GSC hybridized to Applied Biosystems Human Genome Survey Microarray V2.0, scanned using an ABI 1700 Chemiluminescent Microarray Analyzer. The downloaded arrays were quantile normalized and log2-transformed [40]. GSE124145 analyzed the expression patterns of 1 primary IDH-wild type GBM tissue from a 54-year-old female patient, as well as human GSC lines X01 and X03 and the U251 cell line, each in triplicate. The data used the global scaling method to normalize the intensities and was annotated using the same method used for GSE15209 [41]. GSE23806 was a large panel of microarray datasets pre-processed using gcRMA package for R Version 2.3.1 and annotated using Affymetrix HG-U133 Plus 2.0. This profiled 12 GSC cell lines (8 in two different passages) and corresponding 12 original tumors, 7 clonal sublines derived from two GSC lines, 4 monolayer cultures established from the same tumors as GS-lines using standard serum conditions and 32 conventional glioma cell lines. Here, we were interested in the 12 GSC lines that were categorized in two clusters: Cluster 1 with stem cell phenotypes and Cluster 2 with restricted stem-like character and the matched primary tumor samples [42,43]. The normalized data were downloaded from GEO database, and 2-tailed, unpaired t-test was performed between the sample types. The samples were then ranked based on the p-value, and the false discovery rate (FDR) values were calculated. FDR ≤ 0.05 was considered significant and used in further analysis.

#### 4.1.5. Correlation between Genes

To gain a better insight into the roles in stemness, proliferation, differentiation and cell death, we looked for the correlation of the genes with various signature genes associated with the processes in various GBM and GSC datasets mentioned previously.

### 4.2. Enrichment of GBM CSCs

An authenticated U87 cell line, tested for mycoplasma, was procured from NCCS, Pune. The cells were cultured in Minimum Essential Media (MEM) supplemented with 1% antibiotic-antimycotic solution and 10% heat inactivated Fetal Bovine Serum (FBS) and incubated at 37 °C in 5% CO_2_.

The differential trypsinization protocol described by Morata-Tarifa et al. was used for the enrichment process. Cells that were 70–80% confluent were washed twice with PBS and treated with 0.05% trypsin for 2 min. The detached cells were collected, centrifuged and resuspended in fresh media. This was the trypsin-sensitive population (S). The remaining cells were again treated with 0.05% for 4 min and removed to obtain better separation between sensitive and resistant populations. The cells left post treatment were treated with 1X trypsin for 3 min and collected, centrifuged and resuspended in a fresh medium. This was the trypsin-resistant population (R). From another flask seeded at the same time, the entire population of cells were collected using regular trypsinization protocol, and this total population (P) was the control cells in the following assays. All the reported experiments were performed in triplicate.

#### 4.2.1. Sphere Formation Assay

A total of 1000 cells of each type per well were cultured in non-adherent 24-well cell culture plates in serum-free sphere-forming medium (SFM). Dulbecco’s Modified Eagle Medium/Nutrient Mixture F-12 Ham (DMEM/F12) medium (HiMedia-AT127) with 1% antibiotic antimycotic solution, 10 ng/mL recombinant human FGF (Invitogen-PHG0264), 10 ng/mL recombinant human EGF (Invitrogen- PHG0311), 2% B27 supplement (Invitrogen-12587010), was used for this. This was incubated in standard incubating conditions for 6 days, and the medium was replenished depending on growth of the cells. The size and number of spheres were counted after 6 days. The spheres in primary culture were collected, trypsinized and centrifuged. Five hundred cells per well were sub-cultured in SFM with the addition of fresh medium in between. After 6 days, the number of spheres with a size > 70 µm were counted using light microscopy (Olympus CKX42), and the size of spheres formed was analyzed using Image J.

#### 4.2.2. Soft Agar Assay

A 2X concentration of agar and prewarmed media was mixed to form 0.8% base agar, and this was added to each well of the 24-well plate. Once this solidified, 1000 cells per well mixed with 0.4% top agar was seeded, and after incubation for 30 min, fresh medium was added. The medium was supplemented at regular intervals and incubated at 37 °C and 5% CO_2_ for 3 weeks. The number of colonies formed was counted for the different population of cells [59].

#### 4.2.3. Single-Cell Sphere Formation Assay

Cells were plated at a density of 0.5 cell per well. After overnight incubation at 37 °C, the wells with just one cell were scored. Those wells with more than one cell and no cell were excluded. At 10–14 days post seeding, the colony-forming ability from a single cell was observed [60]. The percentage sphere-forming efficiency was determined using $\left(Y\left(n\right)/X\left(n\right)\right)*100$, where *X*(*n*) is the number of wells with single cells as scored on Day 1 and *Y*(*n*) is the number of wells with colonies from a single cell post 14 days incubation.

#### 4.2.4. Proliferation Assay

Same number of cells from P, S and R populations was plated, and after 4 days of growth, the cells were counted using trypan blue staining with 4 mg/mL trypan blue, and the proliferation rate was calculated [61].

#### 4.2.5. Scratch Assay

An equal number of cells of all the populations were plated into 6-well plates and after 48 h of growth, the scratch was made at three different points in each of the wells. The cells were washed with PBS and kept for incubation in a serum starved medium. The movement of cells was observed at 0 h, 12 h, 24 h and 36 h [34]. The area covered was measured using Image J at different time points and migration was indirectly quantified using the area method.

$$\mathrm{percentage}\;\mathrm{wound}\;\mathrm{area}\;\mathrm{at}\;\mathrm{time}\;t=\left(\frac{A\left(t\right)}{A\left(0\right)}\right)\times 100$$

where *A*(*t*) and *A*(0) are the area of the gap covered at any time *t* and at the start of the experiment.

#### 4.2.6. Immunostaining

Five thousand cells per well were seeded on to coverslips, washed in 100% ethanol and placed in 24-well plates. The cells were incubated until they achieved 50–60% confluency. The medium was removed, and the cells were washed with PBS and fixed with 4% paraformaldehyde (PFA) for 10 min. PFA was removed and the cells were washed with wash buffer, tris buffered saline (TBS), and blocked with 1% BSA to prevent non-specific protein binding. The blocking buffer was removed and the cells were washed with TBS. Primary antibodies survivin (sc-17779), SOX2 (Invitrogen-MA1-014) and Nestin (Invitrogen-MA1-110) were added to the cells and were incubated overnight at 4 °C. Staining was visualized with mouse IgGκ conjugated to fluorescein isothiocyanate (FITC), Goat Anti-Mouse IgG(H + L) cross-adsorbed secondary antibody ((Invitrogen -F2761) and (Invitrogen- P-852) and counterstained with DAPI. The specimens were mounted on microscope slides and visualized. Fluorescence observation and photo-documentation were obtained using an inverted fluorescent microscope [62].

### 4.3. Effect of Survivin Inhibition on Isolated GSCs

#### 4.3.1. Cell Viability Assay

We used the MTT [3-(4,5-dimethylthiazol-2-yl)-2,5 diphenyl tetrazolium bromide] assay for determining the cytotoxicity of the drugs, calculating cell viability and studying the effect of combination of the drugs. Five thousand cells per well were plated in 96-well plates. After overnight incubation the cells were treated with 0, 12.5, 25, 50, 100, 200, 400 and 500 μM of both PIP (Sigma-P49007) and TMZ (Caymen-14163). Ethanol and DMSO were used as vehicle control for PIP and TMZ, respectively. After 72 h of incubation, 2 mg/mL freshly prepared MTT (Sigma-M5655) was added and incubated for 4 h. The formazan crystals were then dissolved using DMSO, and the absorbance was taken at 540 nm. The IC50 values of the drugs were calculated using GraphPad-Prism, and concentrations around the IC10 values were taken for further experiments.

#### 4.3.2. Real-Time PCR

RNA isolation was performed by TRIzol (Invitrogen-15596026) method following the manufacturer’s protocol, and quality and yield was measured using Eppendorf Biospectrophotometer Basic. RNA samples with A260/280 ratio ~2 were used for downstream applications. iScript cDNA synthesis kit was used for cDNA synthesis, and 1 µg of RNA per 20 µL of cDNA synthesis reaction was used here. The cDNA synthesized was immediately aliquoted to working volumes and stored at −20 °C. SYBR green (10 µL), primers (350 nM each), water and 20 ng of cDNA was used in each 20 µL of reaction. The program was set as per the manufacturer’s protocol. The annealing temperature for each primer pair was decided based on the primer optimization data. The temperature details are given in Table 1.

#### 4.3.3. Combination Index

To understand the nature of interaction between *PIP* and TMZ on U87 cell lines, we incubated the cells with PIP and TMZ at various rations based on the IC50 values. Here, we used both constant as well as non-constant ratios of the drugs, where the constant ratio of 1:1.5 (PIP:TMZ) and non-constant ratio with PIP were fixed at 25 µM and varying concentrations of TMZ in the ratio (1:1.5, 1:3, 1:6) of PIP and TMZ for 24, 48 and 72 h. MTT assay was performed post-incubation as described before. The combination index (CI) was calculated using CompuSync software [63]. The CI values < 0.8 indicated synergy, 0.8 < CI < 1.2 shows additive effect and CI > 1.2 suggests antagonism.

#### 4.3.4. Clonogenicity

To observe the effects of PIP, TMZ and the combination on the colony-forming potential of these P and S pools, we plated 50 cells per well in SFM and treated with 0, 12.5 µM, 25 µM and 50 µM of PIP, 37.5 µM TMZ and a combination 25 µM PIP and 37.5 µM TMZ and incubated for 14 days. The medium was replenished every 5 days, and colonies formed were analyzed for the number and size using light microscopy.

#### 4.3.5. Invasion Assay

The effect of the treatment on the metastatic potential of the stem cell pool was studied by plating single spheres grown with and without treatment in non-adherent culture conditions on Geltrex (Invitrogen-A1413302). Geltrex is a soluble basement membrane that acts as an extracellular matrix assisting tumor invasion. A total of 50 µL cold Geltrex was gently poured on to each well of a 96-well plate and shifted to 37 °C for allowing gel formation. Single spheres are then transferred onto the gel. A total of 50 µL fresh media was added, subsequent treatment was given and the cells were incubated. The spheres were observed every alternate day, and the movement of the cells was documented using light microscopy. Here, two-way ANOVA was used for statistical analysis.

#### 4.3.6. Acridine Orange-Ethidium Bromide (AO/EtBr) Staining

AO/EtBr staining was used to visualize and quantify live and apoptotic cells. A total of 2 µL AO and EtBr each, at a concentration of 100 µg/mL, were mixed with the trypsinized cells with and without treatment. The cells were incubated for 5 min, and 10 µL of the mix was placed onto a glass slide and observed under fluorescent microscope. Live cells and early apoptotic cells took up AO and appeared green, whereas late apoptotic cells and necrotic cells took up EtBr and appeared red when observed under a fluorescent microscope.

### 4.4. Statistical Analysis

All experiments were performed in triplicate, and the mean and standard deviation (SD) were calculated. All graphed data and statistical significance analysis were performed using GraphPad Prism 5.0 and were normalized to control and reported as mean fold change ± SD. For comparing two groups, Students’ two-tailed t-test was used and for more than two groups, one-way ANOVA followed by Tukey’s multiple comparison test was used. * Denotes *p*-value < 0.05; **: *p*-value < 0.01; ***: *p*-value < 0.001.

## 5. Conclusions

The multifaceted oncofetal protein survivin was found to be differentially expressed in GSCs when compared to hGBM, aNSC and normal brain. We also found it to be correlated with genes responsible for various cancer processes. Human GSC isolated from the U87 cell line showed high expression of survivin. Survivin suppression in these cells using the indigenous dietary phytochemical PIP showed a subsequent reduction in stemness and proliferation. Interestingly, the inhibition increased the sensitivity of the cells to first-line chemotherapy drug TMZ and also induced apoptosis in these cells in combination with TMZ. Our results point towards the significance of survivin in GBM and GSC compartments and its potential as a direct target for GSCs. The inhibitor PIP would be a promising drug for direct targeting of survivin in GSCs. We were able to suggest a new combination treatment modality of PIP with TMZ, for GBM, more precisely for the GSC compartment in GBM.

The future scope of this research would be the validation of this work on GBM animal models with knockout and then clinical trials for establishing the potential of survivin in GSC targeting. Studying survivin at single-cell level in GSCs is important in developing this molecule further as a GSC biomarker. Co-targeting of BIRC5 with various other genes that have significant roles in cancer progression is another avenue that needs to be explored for developing better targeting strategies. The differential expression of the gene across cancer types opens up the avenue of developing it as a CSC marker and needs further in-depth analysis and experimentations.

## Figures and Tables

**Table 1:** The forward and reverse primers used in RT-PCR.

Gene		Primer	Ta
BIRC5	FP	CTGAAGTCTGGCGTAAGATG	56 °C
	RP	AGCGAAGCTGTAACAATCC	
SOX2	FP	AAGGAGCACCCGGATTAT	56 °C
	RP	GCAGCGTGTACTTATCCTTC	
GAPDH	FP	TCGACAGTCAGCCGCATCTT	64 °C
	RP	CGCCCAATACGACCAAATCC	

## Data Availability

All the data used in this study were drawn from public databases and were permitted for use. The comparison of TCGA tumors with GETx normal was performed using GEPIA (http://gepia.cancer-pku.cn/). Expression patterns in various stem cell types were collected from freely available Stemformatics (https://www.stemformatics.org/). The microarray and RNA-seq datasets, used in the expression analysis in GBM and LGG, were obtained from GlioVis (http://gliovis.bioinfo.cnio.es/). Microarray datasets for expression analysis in GSCs, GSE15209, GSE31262, GSE124145 and GSE23806 are from the GEO database (https://www.ncbi.nlm.nih.gov/gds/).

## Supplementary Materials

The following supporting information can be downloaded at: https://www.mdpi.com/article/10.3390/ijms23147604/s1.

## Abbreviations

ABC	ATP-Binding Cassette
ATP	Adenosine Triphosphate
Ang-1	Angiopoietin-1
AO	Acridine Orange
BAX	B-cell Lymphoma 2 Associated X
BBB	Blood−Brain Barrier
BCL2	B-Cell Lymphoma 2
BTB	Blood−Tumor Barrier
BIRC5	Baculoviral IAP Repeat (BIR) Containing 5
BSA	Bovine Serum Albumin
CD	Cluster of Differentiation
CGGA	Chinese Glioma Genome Atlas
CSCs	Cancer Stem Cells
DAPI	4′,6-diamidino-2-phenylindole
EMT	Epithelial Mesenchymal Transition
ESC	Embryonic Stem Cells
EtBr	Ethidium Bromide
FBS	Fetal Bovine Serum
FITC	Fluorescein Isothiocyanate
GAPDH	Glyceraldehyde 3-phosphate dehydrogenase
GBM	Glioblastoma Multiforme
GEO	Gene Expression Omnibus
GFAP	Glial Fibrillary Acidic Protein
GSCs	Glioblastoma Stem Cells
IAP	Inhibitor of Apoptosis Protein
ICC	Immunocytochemistry
IDH	Isocitrate Dehydrogenase
iPSCs	Induced Pluripotent Stem Cells
JAK	Janus Kinase
L1CAM	L1 Cell Adhesion Molecule
LGG	Lower Grade Glioma
MEM	Minimum Essential Media
mTOR	Mammalian Target of Rapamycin
MTT	3-(4,5-dimethylthiazol-2-yl)-2,5-diphenyltetrazolium bromide
NES	Nestin
NF-ĸB	Nuclear Factor Kappa-Light-Chain-Enhancer of Activated B Cells
NSC	Neural Stem Cells
fNSCs	Fetal NSCs
aNSCs	Adult NSCs
Oct4	Octamer-Binding Transcription Factor 4
OLIG2	Oligodendrocyte Transcription Factor 2
PBS	Phosphate-Buffered Saline
PI3K	Phosphoinositide-3 Kinase
PIP	Piperine
PROM1	Prominin 1
RNA-seq	RNA Sequencing
RT-PCR	Reverse Transcriptase Polymerase Chain Reaction
SALL4	Sal-like protein 4
SFM	Serum-Free Media
SMAC	Second Mitochondria-Derived Activator of Caspase
SMAD	Small Mothers Against Decapentaplegic
SOX2	SRY (Sex-Determining Region Y)-Box 2
SSEA	Stage-Specific Embryonic Antigen-4
STAT	Signal Transducer and Activator of Transcription
TBS	Tris-Buffered Saline
TCGA	The Cancer Genome Atlas
TGF-β	Transforming Growth Factor Beta
TMZ	Temozolomide
TUBB3	Tubulin Beta 3 class III
