# Supplementary material for: Survivin Inhibition by Piperine Sensitizes Glioblastoma Cancer Stem Cells and Leads to Better Drug Response
Source: Int J Mol Sci. 2022 Jul 9;23(14):7604. doi: 10.3390/ijms23147604 (PMC9323232; doi:10.3390/ijms23147604)
Supplement: Supplementary file 1 [file ijms-23-07604-s001.zip › ijms-1749739-supplementary.pdf]

Supplementary Files

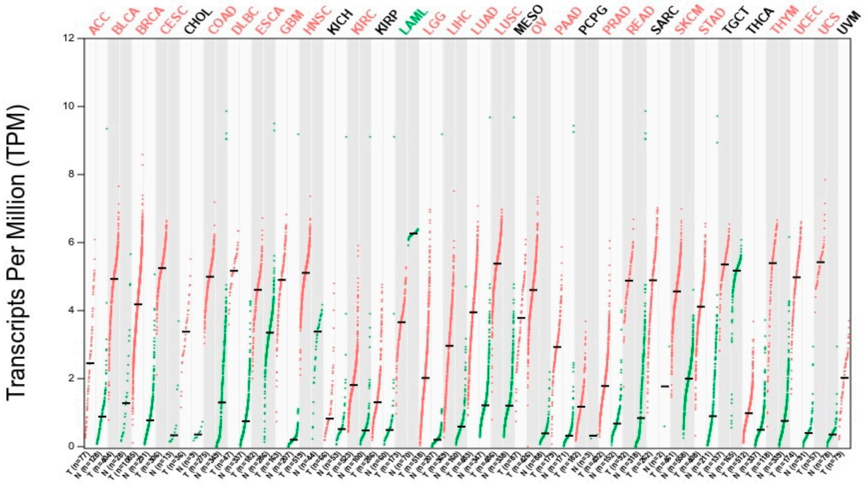

Figure S1a: Differential expression profiles of baculovirus inhibitor of apoptosis (IAP) repeat containing 5 (BIRC5) across 33 cancer types in TCGA and matched normal tissues from GTEx database from GEPIA. The abbreviations for the tumor types are standard TCGA study abbreviations and can be found in at (https://gdc.cancer.gov/resources-tcga-users/tcga-code-tables/tcga-study-abbreviations).

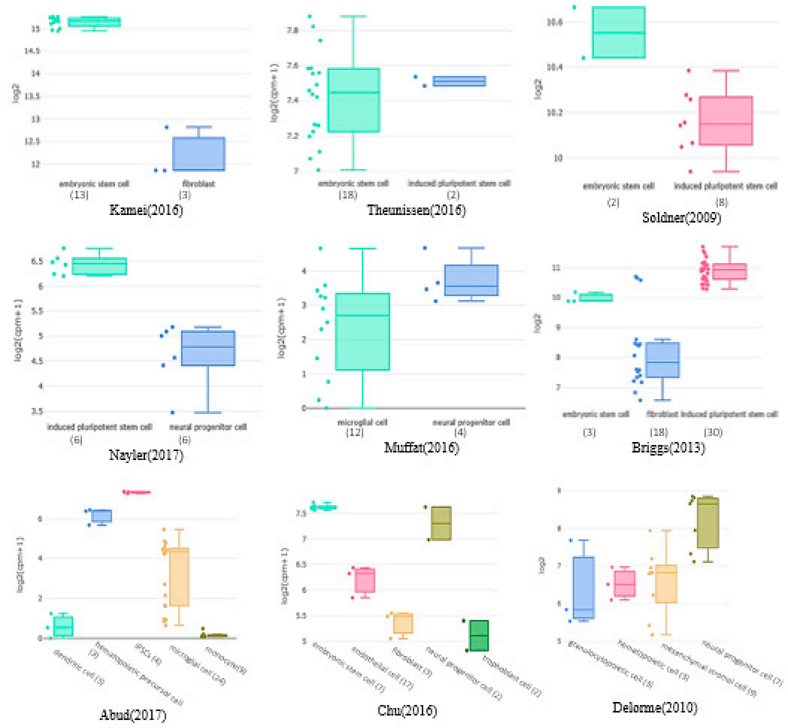

Figure S1b: Expression patterns of BIRC5 in various cell types in 9 different data types obtained from https://www.stemformatics.org/. The name of the dataset and number of samples per cell types are indicated ~~bellow~~below each graph.

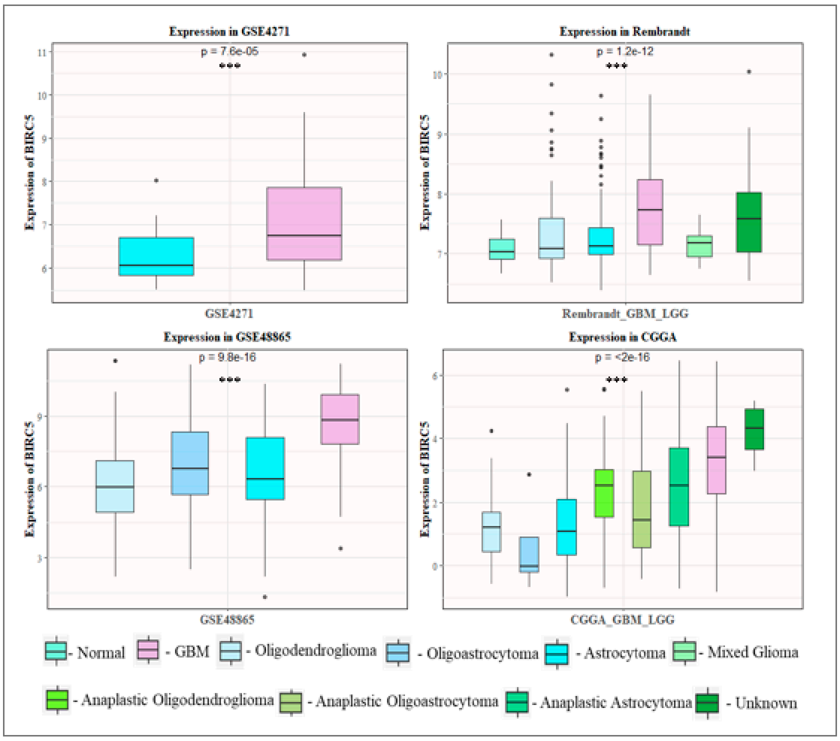

Figure S1c: Differential expression of BIRC5 and g~~G~~lioblastoma (GBM), l~~L~~ower grade gliomas (LGG) and n~~N~~ormal brain. Four~~4~~ d~~D~~ifferent studies from GlioVis comparing GBM, LGG and normal samples~~.~~. The data ~~is~~ are presented as mean ± SD, and the statistical analysis was performed using one-~~-~~way ANOVA (*:*p*-value < 0.05; **: *p*-value <0.01; ***: *p*-value < 0.001).

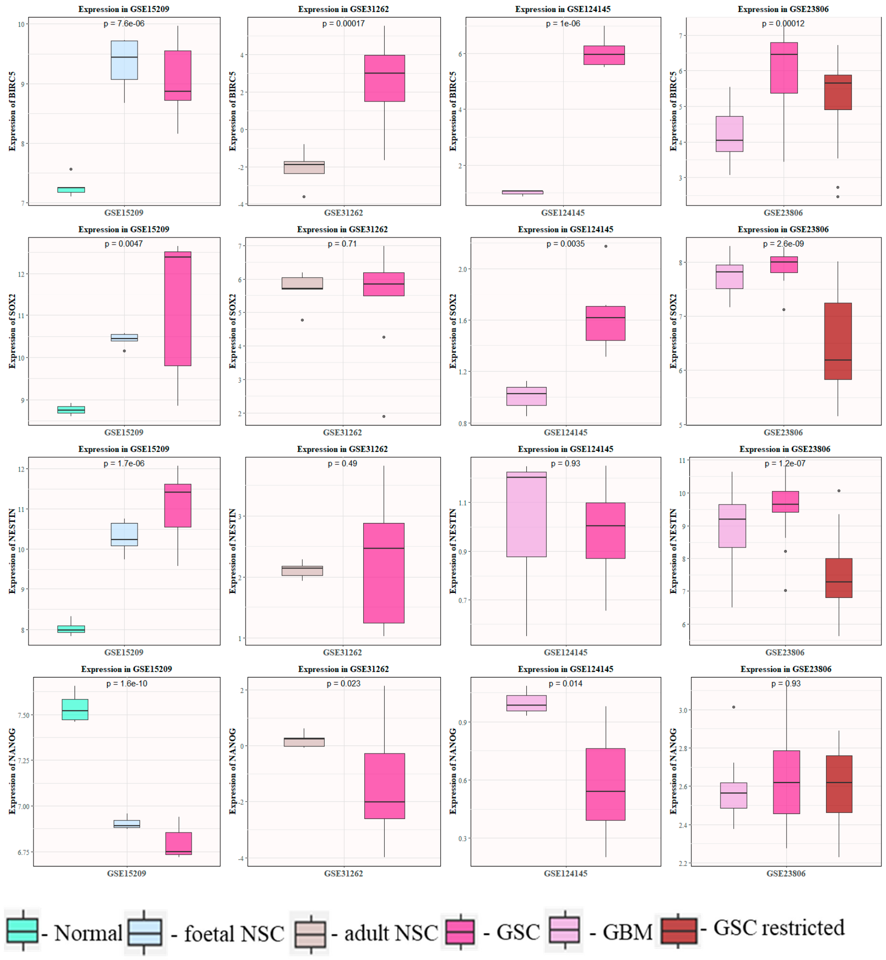

Figure S2: Comparison of the expression profiles of literature-curated stemness markers with BIRC5. The data ~~is~~ are presented as mean ± SD, and the statistical analysis was performed using one-way ANOVA (*: *p*-value < 0.05; **: *p*-value <0.01; ***: *p*-value <0.001).

Formatted: Font: Not Italic

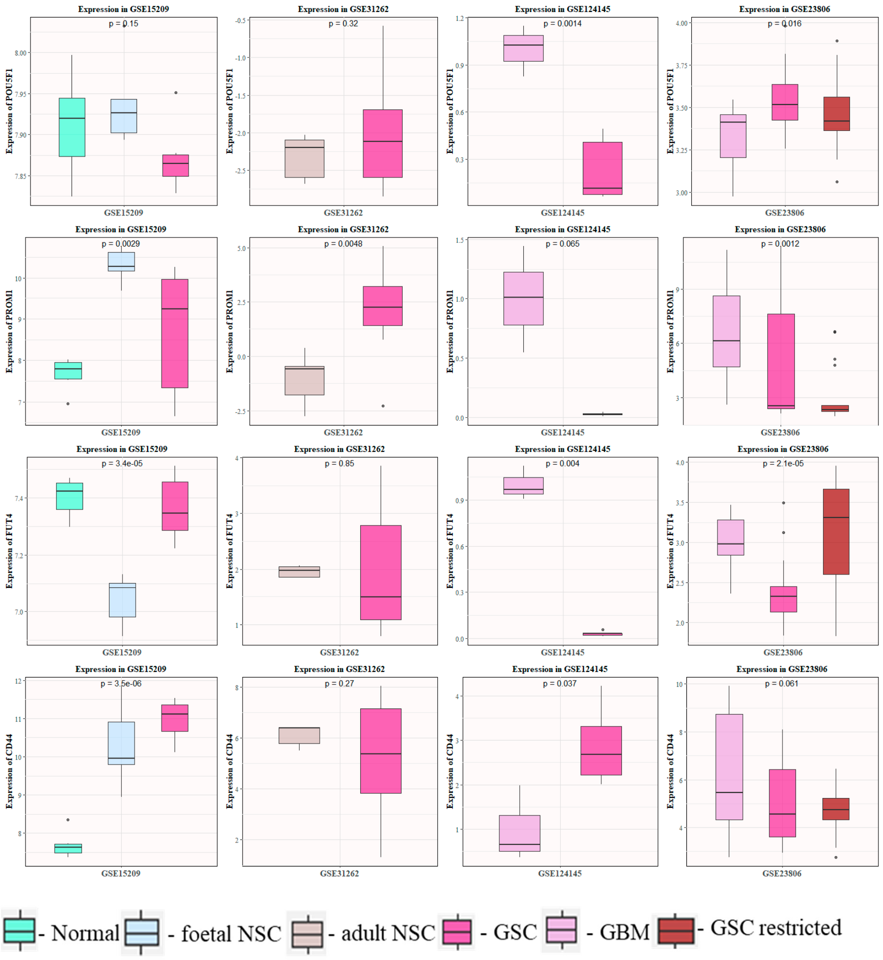

Figure S2: Comparison of the expression profiles of literature-curated stemness markers with BIRC5. The data ~~is~~ are presented as mean ± SD, and the statistical analysis was performed using one-way ANOVA (*:p-value < 0.05; **: p-value <0.01; ***: p-value <0.001).

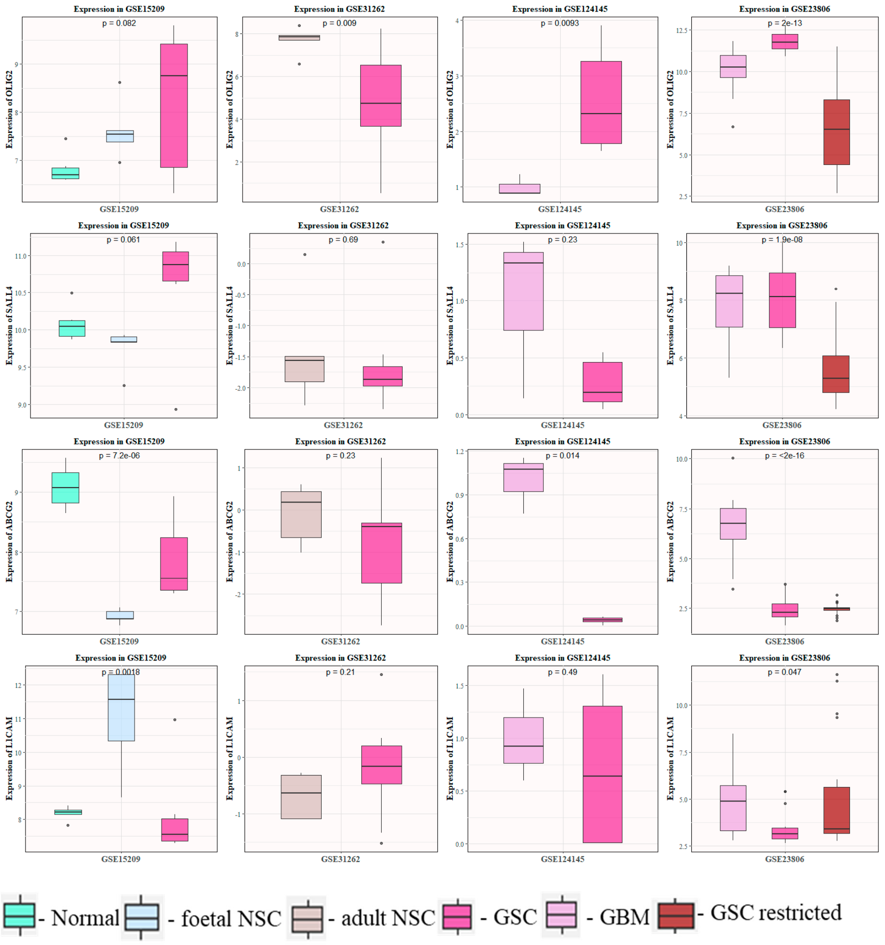

Figure S2: Comparison of the expression profiles of literature-~~-~~curated stemness markers with BIRC5~~.~~. The data ~~is~~ are presented as mean ± SD, and the statistical analysis was performed using one-way ANOVA (*:*p*-value < 0.05; **: *p*-value <0.01; ***: *p*-value <0.001).

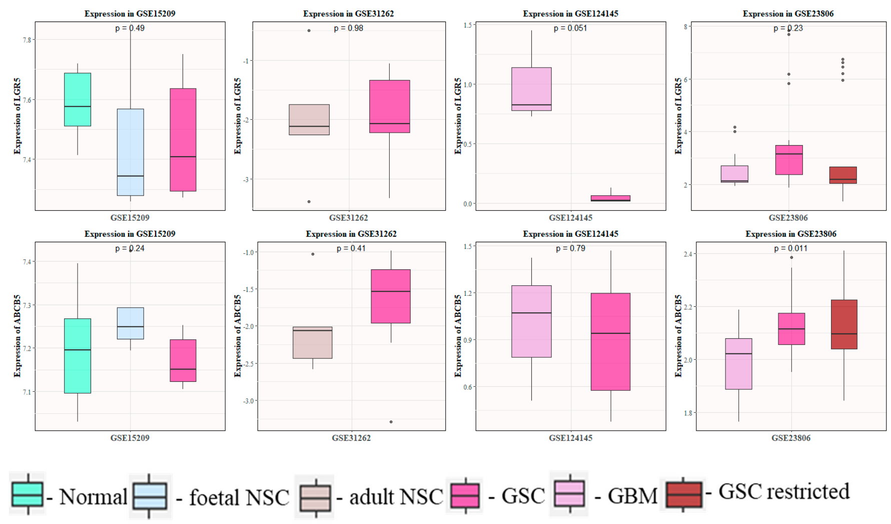

Figure S2: Comparison of the expression profiles of literature-curated stemness markers with BIRC5. The data are presented as mean ± SD, and the statistical analysis was performed using Student's *t*-test and one-way ANOVA (*: *p*-value < 0.05; **: *p*-value <0.01; ***: *p*-value <0.001).

Formatted: Font: Not Italic

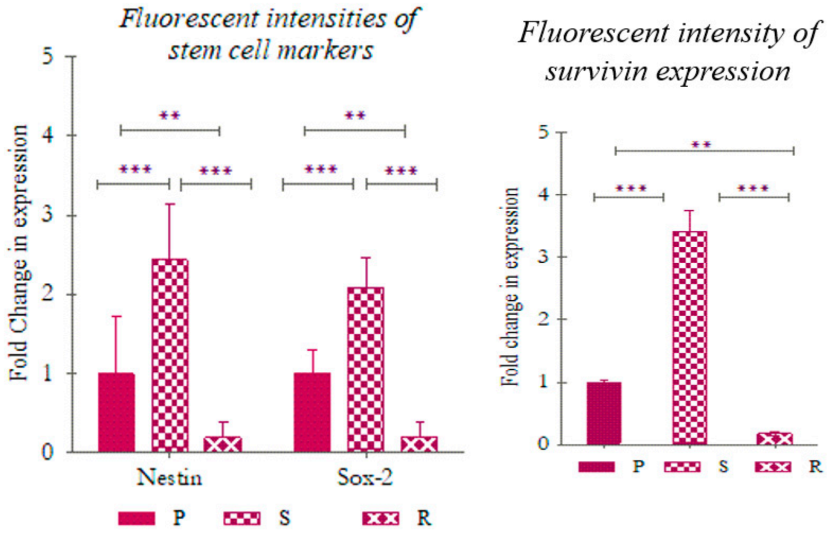

Figure S3: Analysis of fluorescent intensities of staining images. (A) Fold change in expression levels of stem cell markers, Nestin and Sox2. (B) Fold change in expression levels of survivin. The intensities were measured using ImageJ. The data are presented as mean ± SD, and the statistical analysis was performed using one-way ANOVA (*: *p*-value < 0.05; **: *p*-value <0.01; ***: *p*-value <0.001).

Commented [M. C.1]: There is no A and B in the image. Please fix.

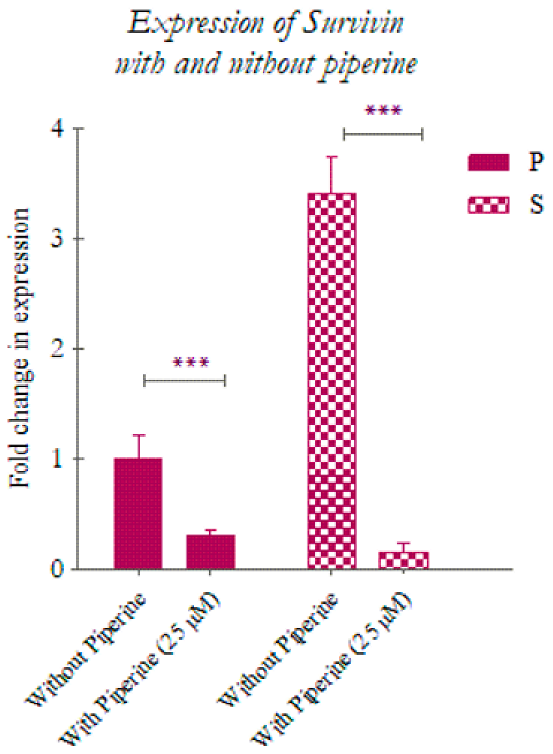

Figure S4: Analysis of fluorescent intensities of staining images showing fold change in expression levels of survivin with and without PIP treatment in P and S cells. The intensities were measured using ImageJ. The data are presented as mean ± SD, and the statistical analysis was performed using one-way ANOVA (*: p-value < 0.05; **: p-value <0.01; ***: p-value <0.001).
